# Tailoring the catalytic performance of Cu/SiO_2_ for hydrogenolysis of biomass-derived 5-hydroxymethylfurfural to renewable fuels

**DOI:** 10.3389/fchem.2022.979353

**Published:** 2022-08-22

**Authors:** Hongyan Jia, Qing Lv, Qineng Xia, Wanpeng Hu, Yanqin Wang

**Affiliations:** ^1^ College of Biological, Chemical Science and Engineering, Jiaxing University, Jiaxing, China; ^2^ Shanghai Key Laboratory of Functional Materials Chemistry, Research Institute of Industrial Catalysis, School of Chemistry and Molecular Engineering, East China University of Science and Technology, Shanghai, China

**Keywords:** 5-hydroxymethylfurfural, 2,5-dimethylfuran, renewable fuels, hydrogenolysis, Cu/SiO2 catalyst

## Abstract

Efficient conversion of biomass-derived 5-hydroxymethylfurfural (HMF) to renewable fuels such as 2,5-dimethylfuran (DMF) and 2,5-dimethyltetrahydrofuran (DMTHF) is of significance for sustainable energy supply. For efficient catalyst design, it is important to understand the catalytic behavior and clarify the influence of physico-chemical properties of catalyst on reaction performance. Herein, to study the structure-activity relationships of monometallic Cu catalysts for HMF hydrogenolysis, a series of Cu/SiO_2_ catalysts with different physico-chemical properties were prepared and compared for their catalytic performance in HMF hydrogenolysis. It was found that Cu/SiO_2_-HT-8.5 catalyst prepared by hydrothermal method showed excellent activity in HMF hydrohydrolysis reaction. Under the optimal reaction condition, the total yield of liquid fuels reaches 91.6% with 57.1% yield of DMF and 34.5% yield of DMTHF in THF solvent. Characterizations such as XRD, H_2_-TPR, N_2_-adsorption/desorption, TEM and XPS revealed that the Cu particles in the Cu/SiO_2_-HT-8.5 catalyst have uniform size and high dispersion. The Cu species and the SiO_2_ support have relatively weak interaction and are easy to be reduced to Cu^0^, which makes it show excellent activity in the hydrogenolysis of HMF.

## Introduction

Efficient conversion of renewable lignocellulosic biomass to bio-fuels and value-added chemicals is of significance for sustainable energy supply, and reduction of CO_2_ emissions, as an alternative strategy to many other ways to solve the energy and environmental issues (Jing et al., 2019; Hao et al., 2021; Qin et al., 2021; Li et al., 2022). 5-hydroxymethylfurfural (HMF) is regarded as one of the most versatile platform molecules that can be converted to a variety of biofuels and chemicals, such as levulinic acid (Yan et al., 2015), liquid alkanes (Xia et al., 2014; Nakagawa et al., 2019), 2,5-furandicarboxylic acid and derivatives (Yang et al., 2020; Nakagawa et al., 2021), 2,5-bis(hydroxymethyl)tetrahydrofuran (BHMTHF) (Nakagawa et al., 2017), 1,6-hexanediol (Xiao et al., 2016) and so forth (Gao et al., 2021). 2,5-dimethylfuran (DMF) and 2,5-dimethyltetrahydrofuran (DMTHF) derived from hydrogenolysis of HMF are not only important chemical intermediates, but also serves as high-grade biofuels with high octane number and high energy density (Roman-Leshkov et al., 2007). Moreover, DMF is also employed as a feedstock for the production of *p*-xylene through the Diels-Alder reaction (Rohling et al., 2018).

The reaction networks for DMF and DMTHF production from HMF contain a series of parallel and consecutive reactions, and therefore it is a challenge to increase the selectivity to the desired products at a complete conversion of HMF (Maki-Arvela et al., 2021). Over the last decade, catalysts based on noble metals (e.g., Pt (Shi et al., 2016), Ru (Zu et al., 2014; Priecel et al., 2018; Feng et al., 2020; Tzeng et al., 2020), Pd (Hu et al., 2021)) and their bimetallics (Requies et al., 2018; Wang et al., 2018; Mhadmhan et al., 2019; Talpade et al., 2019; Pisal & Yadav, 2021) have been extensively explored and proved to be efficient in the hydrogenolysis of HMF to DMF and DMTHF. However, noble metal-based catalysts suffer from the high cost of catalyst preparation, which may limit their large-scale industrial applications.

As an alternative, catalysts based on non-noble transition metals such as Cu, Co and Ni have been widely studied for HMF hydrogenolysis to DMF and DMTHF, with the great majority efforts emphasis on bimetallic catalysts (e.g., Ni-Co (Yang et al., 2015; Yang et al., 2016; Xia et al., 2022), Co-Cu (Guo et al., 2016), Cu-Ni (Zhang Y.-R. et al., 2019; Zhu et al., 2019; Umasankar et al., 2020), Cu-Zn (Zhu et al., 2015; Brzezinska et al., 2020), Ni-Zn (Kong et al., 2017), Co-Fe (Solanki and Rode, 2019) etc.) or bifunctional catalysts that consist of active metal and acidic supports (Shi et al., 2018; Chen N. et al., 2020; Esteves et al., 2020; Guo et al., 2020). For example, Zhu et al. developed an alloyed Cu-Ni encapsulated in carbon catalyst by loading Ni and Cu onto biochar, and the bimetallic Cu-Ni catalyst displayed high catalytic performance for HMF hydrogenolysis to DMF with yield up to 93.5% under the optimized conditions (Zhu et al., 2019). Our group also reported a non-noble bimetallic Ni-Co catalyst with the uniform dispersion of Ni and Co species on the active carbon for hydrogenolysis of HMF, and an excellent yield (up to 95%) of DMF can be obtained at relatively mild conditions (Yang et al., 2016). Very recently, our group reported a unique core-shell structured Co@CoO catalyst for this reaction and afforded the highest productivity among all catalysts reported to date (Xiang et al., 2022).

Earth-abundant Cu-based catalysts are well known for their hydrogenation ability, especially in the field of CO_2_ hydrogenation (Kattel et al., 2017). From the catalyst design point of view, it is important to understand the catalytic behavior and clarify the influence of physico-chemical properties of metal on reaction performance. However, structure-activity relationships of monometallic non-noble metals (Cu, Co, and Ni) on HMF hydrogenolysis have been rarely explored, especially for Cu-based catalysts (Chen S. et al., 2020).

Herein, to study the structure-activity relationships of monometallic Cu catalysts for HMF hydrogenolysis, a series of Cu/SiO_2_ catalysts with different physico-chemical properties were prepared and compared for their catalytic performance in HMF hydrogenolysis. The low acidic non-metal oxide SiO_2_ was chosen as the support to exclude the effect of the acid sites and metal-support interactions as far as possible.

## Experimental

### Chemicals and materials

HMF was purchased from Shanghai Mode Pharmaceutical Technology Co., Ltd. Tetrahydrofuran (THF) was purchased from Shanghai Lingfeng Chemical Reagent Co., Ltd. 2,5-dimethylfuran (DMF), tridecane was obtained from Aladdin Reagent (Shanghai) Co., Ltd. SiO_2_ was obtained from Afaisha Chemical Co. Ltd. All other chemicals and solvents (analytical grade) were purchased from Sinopharm Chemical Reagent Co., Ltd, China. All the chemicals were used without further purification.

### Catalyst preparation

Cu/SiO_2_ catalysts were prepared by three different method, namely excessive impregnation (EI), deposition-precipitation (DP) and hydrothermal (HT) method. For a typical EI method, 1.89 g CuNO_3_•3H_2_O was dissolved in 70 ml deionized water, and then 1.0 g SiO_2_ was added into the solution, stirred at room temperature for 12 h. After that, the suspension was dried at 60°C for 12 h. The obtained precursor was then calcined in static air at 450°C for 4 h, and then reduced in flow 10% H_2_/Ar mixture at 450°C for 6 h before use. The catalyst was denoted as Cu/SiO_2_-EI.

For a typical DP method, 1.89 g CuNO_3_•3H_2_O was dissolved in 70 ml deionized water, and then silica sol (equivalent to 1.0 g SiO_2_) was dropped into the solution and stirred for 0.5 h. Then 1.0 mol/L NaOH solution was dropped into the above mixture with stirring, until the pH of the suspension to a set value of 9. Subsequently, the obtained suspension was filtered, washed by deionized water and dried at 60°C for 12 h. The obtained precursor was then calcined and reduced with the same procedure as EI method. The catalysts were denoted as Cu/SiO_2_-DP.

For a typical HT method, 1.89 g CuNO_3_•3H_2_O was dissolved in 70 ml deionized water, and then a certain amount of NH_4_Cl, NH_3_·H_2_O and silica sol (equivalent to 1.0 g SiO_2_) were added into the solution to reach a set pH value (6.5 or 8.5) and stirred for 0.5 h. After that, the mixture was placed in ultrasonic bath for 0.5 h, and then transferred into a 100 ml Teflon-lined autoclave and aged at 120°C for 4 h. After cooling to room temperature, the suspension was filtered, washed by deionized water and dried at 60°C for 12 h. The obtained precursor was then calcined and reduced with the same procedure as EI method. The catalysts were denoted as Cu/SiO_2_-HT-6.5 or Cu/SiO_2_-HT-8.5.

### Catalyst characterization

The X-ray diffraction (XRD) patterns of the catalysts were recorded by a Bruker D8 Foucus X-ray diffractometer with Cu Kα radiation (*λ* = 0.1541 nm) operating at 40 kv and 40 mA. The crystal size was calculated using the Scherrer equation through the Cu 111) diffraction peak.

N_2_ adsorption-desorption isotherms were measured at −196°C using a Micromeritics ASAP 2020 instrument. The samples were outgassed at 200°C for 6 h before analysis. The specific surface areas were obtained by the Brunauer-Emmett-Teller (BET) method.

H_2_ temperature programmed reduction (H_2_-TPR) was performed on a Huasi DAS-7200 automatic chemisorption apparatus. The samples were pretreated with pure Ar at 150°C for 1 h before reduction. After cooling to room temperature, 10% H_2_/Ar mixture was introduced and the sample tube was heated to 800°C at a heating rate of 10°C/min. TCD was used as the detector to collect signals.

Transmission electron microscopy (TEM) images were taken using a JEOL 2100F microscope operated at an acceleration voltage of 200 kV. The sample was grinded and dispersed in ethanol under supersonic waves, and then dropped on a copper net and dried before use.

The copper content of the catalyst was determined by Agilent 725ES inductively coupled plasma atomic emission spectrometer (ICP-AES).

X-ray photoelectron spectroscopy (XPS) was tested on a Thermo Scientific Escalab 250Xi X-ray photoelectron spectrometer with a monochromatic Al Kα radiation (1,486.6 eV photons). All of the binding energies were calibrated by C 1s and the binding energy of C-C is 284.6 eV.

### Hydrogenolysis reactions

The hydrogenolysis of HMF was carried out in a batch reactor equipped with a magnetic stirrer. Typically, 0.05 g HMF, 0.05 g catalyst, 100 μL tridecane (internal standard) and 5 ml tetrahydrofuran (THF) were put into a Teflon-lined stainless-steel autoclave (50 ml). After purging with H_2_ for three times, the reactor was conducted under H_2_ pressure at the required condition for a desired time with magnetic stirring. After the reaction, the reactor was cooled with ice-water and depressurized carefully. After centrifugation of the catalyst, the liquid products were identified by GC-MS (Agilent 7,890A-5975C) equipped with an HP-5 capillary column and quantitatively analyzed by GC (Agilent GC7890 B) equipped with a flame ionization detector (FID) and an HP-5 capillary column using tridecane as an internal standard. The injector temperature was set at 270°C, and the column temperature was increased from 50 to 200°C with a ramp rate of 10°C/min.

## Results and discussion

### Characterization of catalysts

**FIGURE 1:**
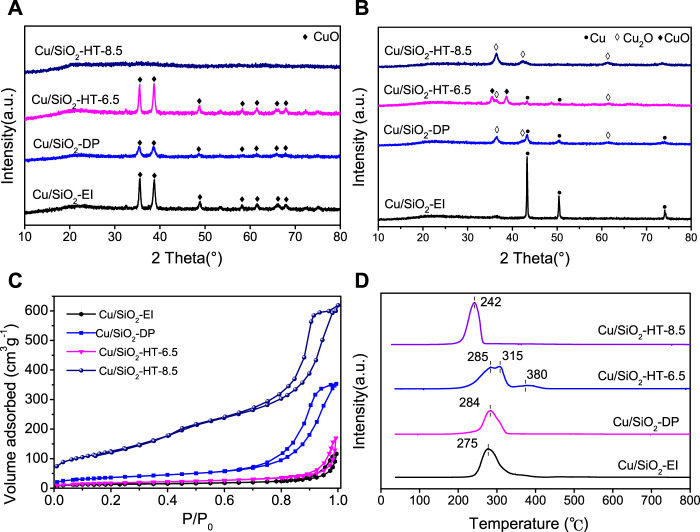
Physicochemical characterization of Cu/SiO_2_ catalysts prepared by different method. **(A)** XRD patterns of Cu/SiO_2_ catalysts before reduction; **(B)** XRD patterns of Cu/SiO_2_ catalysts after reduction; **(C)** N_2_ adsorption-desorption isotherm of Cu/SiO_2_ catalysts; **(D)** H_2_-TPR profiles of Cu/SiO_2_ catalysts.

The Cu/SiO_2_ catalysts were prepared by impregnation (EI), deposition-precipitation (DP) and hydrothermal (HT) methods at different pH value. Figure 1A shows the XRD patterns of various catalysts after calcination. It can be seen that the Cu/SiO_2_-EI and Cu/SiO_2_-DP catalysts exhibit diffraction peaks at 35.5°, 38.7°, 48.7°, 53.5°, 61.5° and 66.2°, indexed to (110), (11–1), (111), (20–2), (020) and (11–3) planes of CuO, suggesting the presence of bulk CuO. Interestingly, obvious CuO diffraction peaks were observed for Cu/SiO_2_-HT-6.5, but none of them was found for Cu/SiO_2_-HT-8.5, which indicates CuO species were highly dispersed on the latter catalyst. The high dispersion of CuO species on Cu/SiO_2_-HT-8.5 can be resulted from the calcination of layered copper silicate (Li et al., 2018). These results indicate that the hydrothermal method favors the Cu^2+^ to complex with surface silanol to form layered copper silicate, leading to high dispersed CuO species after calcination.

Figure 1B shows the XRD patterns of various Cu/SiO_2_ catalysts after reduction. Evident peaks at 43.4°, 50.5° and 74.1° were observed for Cu/SiO_2_-EI catalyst, assigned to (111), (200) and (220) planes of Cu (Dong et al., 2016). The particle size of Cu of Cu/SiO_2_-EI was calculated to be ca. 50 nm according to the Cu 111) diffraction peak, which suggests an agglomeration of Cu cluster. The Cu characteristic diffraction peaks can also be clearly found on Cu/SiO_2_-DP and Cu/SiO_2_-HT-6.5 catalysts, while no obvious Cu diffraction peaks observed on Cu/SiO_2_-HT-8.5, indicating the high dispersion of Cu nanoparticles on the latter catalyst. Cu/SiO_2_-HT-6.5 still exhibits the diffraction peaks at 35.5° (110) and 38.8° (11–1) of CuO, suggesting an incomplete reduction of the catalyst. In addition, Cu/SiO_2_-DP and Cu/SiO_2_-HT catalysts all exhibit the diffraction peaks at 36.5°, 42.3° and 61.3°, attributed to the (111), (200) and (220) planes of Cu_2_O (Wang et al., 2002), indicating the partial reduction of CuO on these catalysts, due to the interactions between CuO_x_ and SiO_2_.

N_2_ adsorption/desorption isotherms for the four Cu/SiO_2_ catalysts are presented in Figure 1C, and the calculated textural properties as well as ICP-AES results are listed in Table 1. All catalysts present the isotherm shape of type IV (IUPAC classification), indicating the existence of mesoporous structure in all cases. There are distinct differences of the textural properties in these catalysts. Cu/SiO_2_-HT-8.5 possesses the largest BET specific surface area (449 m^2^g^−1^) and pore volume (0.92 cm^3^g^−1^), while Cu/SiO_2_-EI shows the smallest ones (48 m^2^g^−1^ and 0.09 cm^3^ g^−1^, respectively). The large BET specific surface area of Cu/SiO_2_-HT-8.5 allows the high dispersion of CuO_x_ species on the SiO_2_ surface, consistent with the XRD results. The Cu contents in Cu/SiO_2_-EI and Cu/SiO_2_-HT catalysts analyzed by ICP-AES were close to the set value (33 wt%), whereas only 23 wt% Cu was detected in Cu/SiO_2_-DP catalyst, which indicates almost a third of Cu content was lost during the DP process.

**TABLE 1:** Cu loading and physical properties of Cu/SiO_2_ catalysts prepared by different method.

Catalyst	Cu loading (wt%)	S_BET_ (m^2^g^−1^)	V_pore_ (cm^3^g^−1^)	Pore size (nm)
Cu/SiO_2_-EI	32	48	0.09	7.6
Cu/SiO_2_-DP-9	23	132	0.52	15.9
Cu/SiO_2_-HT-6.5	33	65	0.12	7.5
Cu/SiO_2_-HT-8.5	31	449	0.92	8.2

The reduction features of Cu/SiO_2_ catalysts were investigated by H_2_-TPR experiments. As shown in Figure 1D, the H_2_-TPR profile of Cu/SiO_2_-EI and Cu/SiO_2_-DP presents a broad hydrogen consumption peak centered at 275 and 284°C, respectively, which can be attributed to the reduction of bulk CuO (Mo and Kawi, 2014). Significant difference was presented for the reduction peaks of Cu/SiO_2_-HT prepared at different pH values. Cu/SiO_2_-HT-6.5 catalyst exhibits two adjacent reduction peaks at 250∼350°C, assigned to the reduction of bulk CuO with multiple sizes. Generally, the reduction peak at high temperature is caused by the reduction of large bulk CuO, and that at low temperature is caused by the reduction of highly dispersed isolated CuO particles (Mo and Kawi, 2014). Cu/SiO_2_-HT-8.5 shows a single symmetrical reduction peak at 242°C. This indicates that there are well-dispersed CuO species with uniform size on the catalyst, which is consistent with the XRD results. Moreover, different reduction temperature can also reflect the strength of the metal-support interactions (Cui et al., 2016). Cu/SiO_2_-HT-8.5 exhibit the lowest reduction temperature of CuO, suggesting the weakest interaction between Cu species and SiO_2_.

**FIGURE 2:**
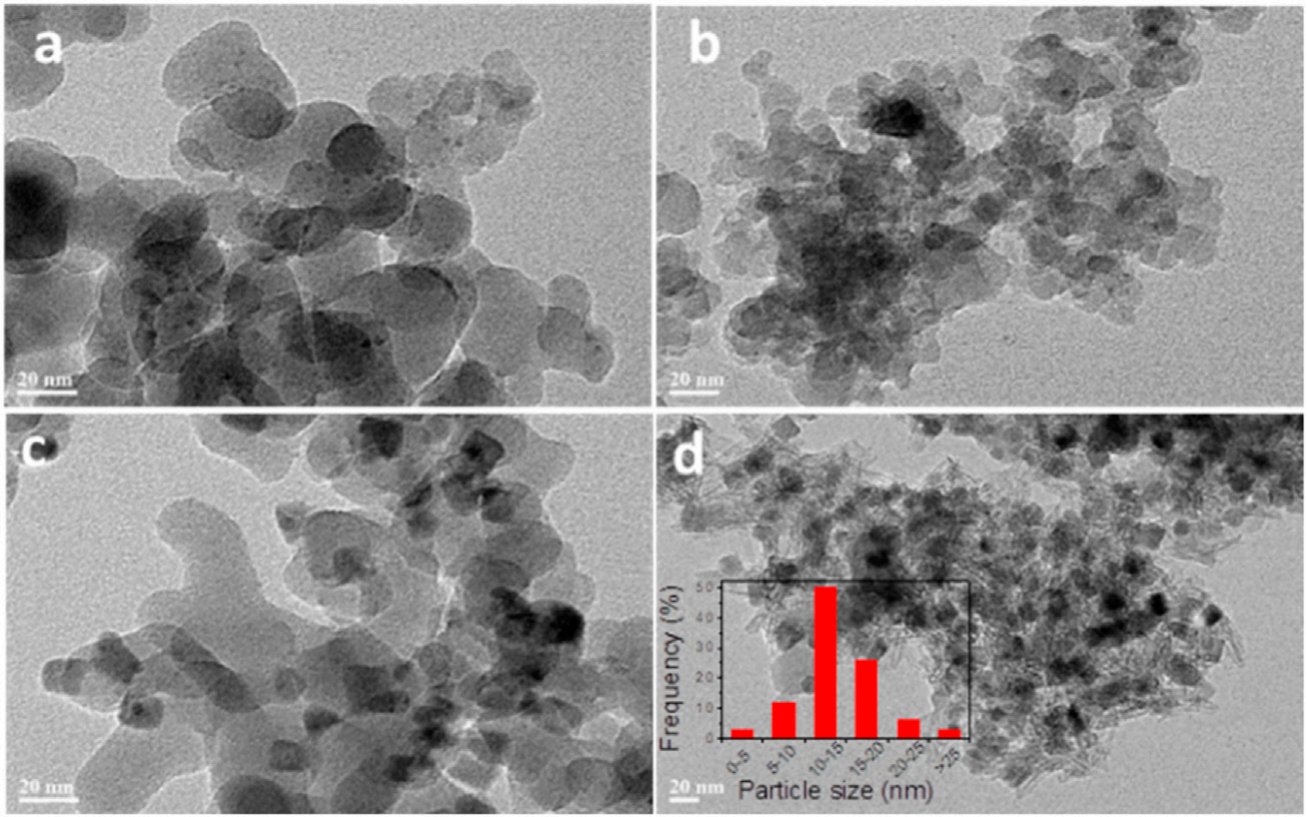
Typical TEM images of Cu/SiO_2_ catalysts prepared by different method after reduction. **(A)** Cu/SiO_2_-EI, **(B)** Cu/SiO_2_-DP, **(C)** Cu/SiO_2_-HT-6.5, **(D)** Cu/SiO_2_-HT-8.5.

The dispersions of the Cu particles in Cu/SiO_2_ catalysts after reduction were further evaluated by TEM (Figure 2). It can be seen from Figures 2A–C that Cu particles were unevenly dispersed on SiO_2_ surface for Cu/SiO_2_-EI, Cu/SiO_2_-DP and Cu/SiO_2_-HT-6.5, with the co-existence of both small and large Cu particles. In contrast, it can be clearly seen that Cu particles are uniformly dispersed on the SiO_2_ support for Cu/SiO_2_-HT-8.5 (Figure 2D). These results are in well agreement with the XRD and H_2_-TPR results. Most of the Cu particles are in the rage of 10–15 nm for Cu/SiO_2_-HT-8.5, as shown in the particle size distribution in Figure 2D. It should be noted that such size (10–15 nm) of Cu particle is relatively small compared to its ultra high Cu loading of 33 wt%. The results reveal that the metal-support interaction adjusted by different preparation method can affect not only the reduction of CuO, but also the dispersion of Cu particles on SiO_2_ surface.

**FIGURE 3:**
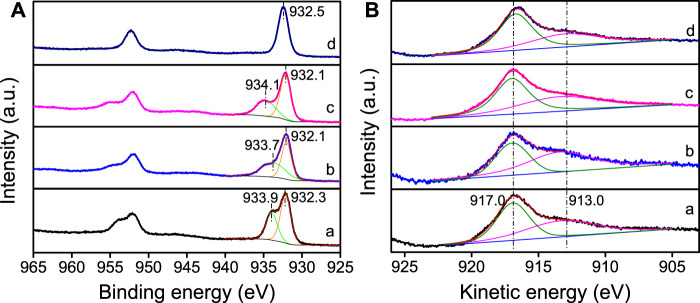
Cu 2p XPS **(A)** and Cu LMM XAFS **(B)** profiles of Cu/SiO_2_ catalysts prepared by different method after reduction. (a) Cu/SiO_2_-EI, (b) Cu/SiO_2_-DP, (c) Cu/SiO_2_-HT-6.5, (d) Cu/SiO_2_-HT-8.5.

The chemical state of Cu species plays a crucial role on the catalytic performance of Cu/SiO_2_ catalysts for HMF hydrogenolysis. The Cu^0^ and Cu^+^ species on the catalyst surface play different roles in catalytic reactions, such as activation of H_2_ or adsorption of substrate. There are two steps of reaction in HMF hydrogenolysis to DMF, namely the hydrogenation of the side -CH = O groups and the following hydrogenolysis of -CH_2_-OH groups, which both need the activation of H_2_ and adsorption of HMF (Zheng et al., 2017). Therefore, XPS and XAES analyses were performed to evaluate the chemical state of Cu species on SiO_2_ surface. As XPS spectra shown in Figure 3A, all four catalysts display binding energy peaks of Cu 2p_3/2_ at 932.1∼932.5 eV, which can be attributed to the formation of Cu^0^ or Cu^+^ (Liu et al., 2015; Li et al., 2016). In the Cu/SiO_2_-EI, Cu/SiO_2_-DP and Cu/SiO_2_-HT-6.5 catalysts, Cu 2p_3/2_ has both low binding energy (932.1∼932.3) and high binding energy (933.7∼934.1), indicating that Cu element in these three catalysts exists not only as Cu^+^ or Cu^0^, but also as Cu^2+^ species (Li et al., 2016). In contrast, no high binding energy peak of Cu 2p_3/2_ was observed in Cu/SiO_2_-HT-8.5 catalyst, attributed to the disappearance of Cu^2+^ species after reduction. These XPS results indicate the highest reducibility of Cu/SiO_2_-HT-8.5, which is in well accordance with H_2_-TPR results.

Since it is difficult to distinguish the Cu^0^ and Cu^+^ by XPS spectra, XAES was carried out to determine the surface Cu^0^ and Cu^+^ species of the reduced Cu/SiO_2_ catalysts. All the reduced catalysts exhibit the Auger kinetic energy peaks of Cu LMM at 917.0 and 913.0 eV (Figure 3B), corresponding to Cu^0^ and Cu^+^ species, respectively (Zhang Z. et al., 2019). According to the peak area of the kinetic energy peak of Cu LMM XAES spectra, we can calculated the ratio of Cu^0^/(Cu^0^+ Cu^+^) (Table 2). The peak area ratio of surface Cu^0^/(Cu^0^+ Cu^+^) varies from 0.40 to 0.59 as the different synthetic method of Cu/SiO_2_, with the Cu/SiO_2_-HT-8.5 catalyst showing the largest proportion of Cu^0^. It is known that the weaker interaction between Cu species and the support leads to the easier reduction of CuO to Cu^0^, while the stronger interaction generates more Cu^+^ species. The largest proportion of Cu^0^ in Cu/SiO_2_-HT-8.5 catalyst indicates that the weakest metal-support interaction compared with the other three catalysts, which is consistent with the H_2_-TPR results.

**TABLE 2:** Kinetic energy and ratio of Cu^0^/(Cu^0^+ Cu^+^) of Cu/SiO_2_ catalysts prepared by different method.

Catalyst	KE^a^ (eV)		Cu^0^/(Cu^0^+ Cu^+^)^b^
	Cu^+^	Cu^0^	
Cu/SiO_2_-EI	913.4	916.9	0.51
Cu/SiO_2_-DP	913.4	917.0	0.40
Cu/SiO_2_-HT-6.5	913.4	917.0	0.54
Cu/SiO_2_-HT-8.5	913.0	916.7	0.59

a Kinetic energy.

b The ratio of Cu^0^/(Cu^0^+ Cu^+^) was calculated from Cu LMM XAES spectra.

### Catalytic performances for hydroxymethylfurfural hydrogenolysis

The reaction activity of Cu/SiO_2_ catalysts prepared by different methods in HMF hydrogenolysis is shown in Table 3. HMF was completely converted in all cases, indicating the hydrogenation of HMF to BHMF is easy over Cu-based catalysts. However, the selectivity of the products was obviously different. Among them, the catalyst prepared by over-impregnation method (Cu/SiO_2_-EI) and deposition precipitation method (Cu/SiO_2_-DP) gave 2,5-dihydroxymethylfuran (BHMF) as a main product with 83.1 and 84.9% yield, respectively, while the total yields of 2.5-dimethylfuran (DMF) and further hydrogenation product 2.5-dimethyltetrahydrofuran (DMTHF) were less than 10%, suggesting a poor hydrogenolysis activity. In contrast, Cu/SiO_2_ prepared by hydrothermal method offered a much stronger hydrogenolysis activity with DMF and DMTHF as the majority products, especially for Cu/SiO_2_-HT-8.5 which achieving the highest activity in HMF hydrohydrolysis with 57.1% yield of DMF and 34.5% yield of DMTHF. It was reported that the activity of Cu-based catalysts in HMF hydrogenolysis is proportional to the specific surface area of the metallic Cu^0^. The high activity for HMF hydrogenolysis over Cu/SiO_2_-HT-8.5 catalyst can be attributed to the high Cu dispersion, small Cu particle size and high proportion of Cu^0^, which is well evidenced by XRD characterization, H_2_-TPR, TEM images, XPS and XAES spectra.

**TABLE 3:** Catalytic performance of HMF hydrogenolysis over Cu/SiO_2_ catalysts prepared by different methods.

Catalysts	Conv. of HMF(%)	Yield (%) or selectivity (%)				
		DMF 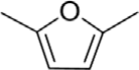	DMTHF 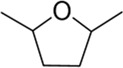	BHMF 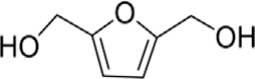	HMMF 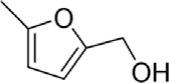	Carbon balance
Cu/SiO_2_-EI	>99.9	5.0	1.6	83.1	7.6	97.3
Cu/SiO_2_-DP	>99.9	3.4	0.5	84.9	7.8	96.6
Cu/SiO_2_-HT-6.5	>99.9	42.9	41.4	0	5.0	90.6
Cu/SiO_2_-HT-8.5	>99.9	57.1	34.5	0	0	93.6

Reaction condition: 0.05 g HMF, 0.05 g catalyst, 100 µL tridecane, 5 ml THF, 200°C, 8 h.

### Reaction pathway study and reaction optimization for hydroxymethylfurfural hydrogenolysis

**FIGURE 4:**
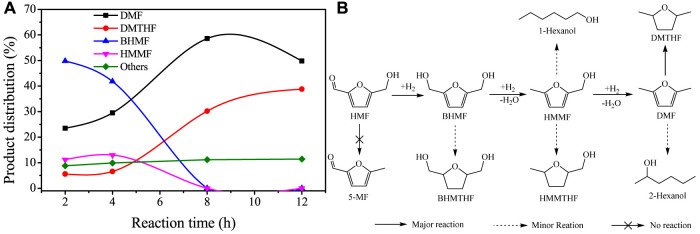
Reaction pathway study of HMF hydrogenolysis over Cu/SiO_2_-HT-8.5 catalyst. **(A)** Product distribution of HMF hydrogenolysis at different reaction time; **(B)** Proposed reaction pathways for HMF hydrogenolysis over Cu/SiO_2_-HT-8.5 catalyst. Reaction conditions: 0.05 g HMF, 0.05 g catalyst, 100 µL tridecane, 5 ml THF, 200°C, 0.6 MPa.

The reaction pathway of HMF hydrogenolysis over Cu/SiO_2_-HT-8.5 was studied by analyzing the product distribution at different reaction time, as shown in Figure 4. It can be seen that HMF has been completely converted when reaction for 2 h, along with a large number of intermediates BHMF (49.8%) and HMMF (11.2%), as well as 23.5% yield of product DMF. This result indicated that the hydrogenation of exocyclic aldehyde group (C=O bond) is a rapid and facile step over Cu/SiO_2_-HT-8.5 catalyst, confirming the high hydrogenation activity of Cu species. It is noted that no 5-methylfurfural (5-MF) was detected, revealing that HMF was first undergo hydrogenation of aldehyde group to BHMF rather than hydrogenolysis of hydroxyl group to 5-MF. After reaction for 4 h, the yield of DMF gradually increased to 29.8%, with the decrease of BHMF to 41.8% and slight increase of HMMF to 13.0%. Only trace of DMTHF was produced at this time, indicating that the hydrogenolysis of hydroxyl group is much faster than the hydrogenation of furan ring over Cu/SiO_2_-HT-8.5. As the reaction further extended to 8 h, the yield of DMF continued to increase to 58.6%, with the complete conversion of BHMF and HMMF. At the same time, it is noteworthy that the yield of DMTHF also increased rapidly to 30.2%, as well as a small amount of hydration ring opening products of DMF. When the reaction was further extended to 12 h, the yield of DMF decreased from 58.6 to 49.8%, and the yield of DMTHF further increased to 38.8%, indicating that the further extension of reaction time would promote the cyclic hydrogenation reaction of DMF to DMTHF. During the reaction period, small amount of other by-products (such as BHMTHF, HMMTHF, hexanols etc., denoted by others) were also detected, totally account for ca. 10%。According to the variation tendency of product distribution versus reaction time, the reaction pathways of HMF hydrogenolysis to DMF and DMTHF over Cu/SiO_2_-HT-8.5 was proposed in Figure 4B.

To further tailor the catalytic performance of Cu/SiO_2_ for HMF hydrogenolysis, we investigated the influence of reaction temperature and H_2_ pressure on the reaction performance, as shown in Table 4. When increase the reaction temperature from 180 to 220^o^C at 0.6 MPa H_2_ pressure, the yield of DMF first increased from 47.5 to 57.1% and then decreased to 51.0%. According to the detection of the product distribution, lower temperature (180^o^C) led to the incomplete conversion of intermediate HMMF and higher temperature (220^o^C) resulted in a small amount of side reaction of DMF to hydration by-products. At the same time, the yield of DMTHF continuously increased from 30.7 to 36% with the increase of reaction temperature, indicating that higher temperature accelerated the reaction rate of furan ring hydrogenation and in favor of the production of DMTHF.

**TABLE 4:** The effects of temperature and pressure on the conversion of HMF over Cu/SiO_2_-HT-8.5 catalyst.

Entry	T/°C	P/MPa	Conv. of HMF	Y_DMF_	Y_DMTHF_	Y_DMF+DMTHF_
1	180	0.6	>99.9	47.5	30.7	88.2
2	200	0.6	>99.9	57.1	34.5	91.6
3	220	0.6	>99.9	51.0	36.0	87.0
4	200	0.2	>99.9	66.8	24.3	91.1
5	200	1.0	>99.9	58.1	35.4	93.5
6	200	1.4	>99.9	46.5	34.4	80.9

Reaction conditions: 0.05 g HMF, 0.05 g catalyst, 100 µL tridecane, 5 ml THF, 8 h.

When the reaction temperature was fixed at 200^o^C, the variation of H_2_ pressure showed that the H_2_ pressure for HMF hydrogenolysis can be as low as 0.2 MPa, at which a high DMF yield of 66.8% was reached, along with 24.3% yield of DMTHF. This result further confirms the high activity of Cu/SiO_2_-HT-8.5 catalyst for HMF hydrogenolysis. Increasing of the H_2_ pressure from 0.2 to 1.4 MPa led to the decrease of the DMF yield from 66.8 to 46.5% with a slight increase of DMTHF, indicating that high H_2_ pressure in favor of the hydrogenation of furan ring, leading to a decline of DMF yield.

### Stability test

**FIGURE 5:**
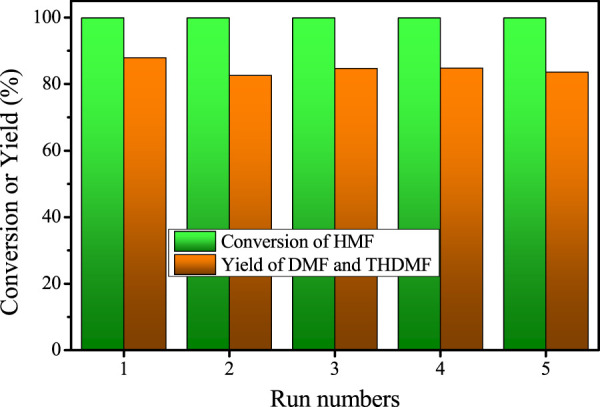
The cycle stability test for HMF hydrogenolysis over Cu/SiO_2_-HT-8.5 catalyst. Reaction conditions: 0.05 g HMF, 0.05 g catalyst, 100 µL tridecane, 5 ml THF, 200°C, 0.6 MPa, 8 h.

The stability of a catalyst is an important factor to evaluate its prospect for industrial application. Therefore, the cycle stability of Cu/SiO_2_-HT-8.5 catalyst for HMF hydrohydrolysis was also investigated under the optimal reaction conditions (200^o^C, 0.6 MPa, 8 h). After the first run, the catalyst was separated from the liquid phase by centrifugation, washed with THF, dried and re-used directly for the second run. No obvious activity loss was found after five successive runs and the total yield of DMF and DMTHF maintain close to the values as the first run (Figure 5), revealing the high stability and recyclability of Cu/SiO_2_-HT-8.5 catalyst. These results suggest that the high dispersion of Cu species with smaller Cu particle offers not only high activity for HMF hydrogenolysis, but also high catalyst stability.

## Conclusion

In summary, we have studied the structure-activity relationships of four Cu/SiO_2_ catalysts prepared by different method for HMF hydrogenolysis. It was demonstrated that Cu/SiO_2_-HT-8.5 catalyst prepared by hydrothermal method showed the best catalytic activity in HMF hydrohydrolysis reaction. Under the optimal reaction condition, the total yield of liquid fuels reaches 91.6% with 57.1% yield of DMF and 34.5% yield of DMTHF in THF solvent. A combination of multiple characterization revealed that the Cu particles in the Cu/SiO_2_-HT-8.5 catalyst have uniform size and high dispersion. The Cu species and the SiO_2_ support have relatively weak interaction and are easy to be reduced to Cu^0^, which makes it an excellent catalyst for the hydrogenolysis of HMF. This work provides a new possibility for cheap monometallic catalyst design for biomass valorization.

## Data Availability

The original contributions presented in the study are included in the article/supplementary material, further inquiries can be directed to the corresponding authors.
